# Diagnostic Validity and Reliability of Low-Dose Prospective ECG-Triggering Cardiac CT in Preoperative Assessment of Complex Congenital Heart Diseases (CHDs)

**DOI:** 10.3390/children9121903

**Published:** 2022-12-04

**Authors:** Yassir Edrees Almalki, Mohammad Abd Alkhalik Basha, Sharifa Khalid Alduraibi, Khalaf Alshamrani, Mohammed Ayed Huneif, Alaa Khalid Alduraibi, Sultan A. Almedhesh, Hassan A. Alshamrani, Khaled Ahmed Ahmed Elbanna, Youssef H. Algazzar, Maha Ibrahim Metwally

**Affiliations:** 1 Division of Radiology, Department of Internal Medicine, Medical College, Najran University, Najran 61441, Saudi Arabia; 2 Department of Diagnostic Radiology, Faculty of Human Medicine, Zagazig University, Zagazig 44519, Egypt; 3 Department of Radiology, College of Medicine, Qassim University, Buraidah 52571, Saudi Arabia; 4 Department of Radiological Science, College of Applied Medical Science, Najran University, Najran 61441, Saudi Arabia; 5 Department of Pediatrics, College of Medicine, Najran University, Najran 61441, Saudi Arabia; 6 Department of Internal Medicine, Faculty of Human Medicine, Zagazig University, Zagazig 44519, Egypt; 7 Department of internal medicine, Katai Gabor hospital, 5300 Karcag, Hungary

**Keywords:** low-dose, prospective ECG-gated, cardiac CT, complex CHDs

## Abstract

For the precise preoperative evaluation of complex congenital heart diseases (CHDs) with reduced radiation dose exposure, we assessed the diagnostic validity and reliability of low-dose prospective ECG-gated cardiac CT (CCT). Forty-two individuals with complex CHDs who underwent preoperative CCT as part of a prospective study were included. Each CCT image was examined independently by two radiologists. The primary reference for assessing the diagnostic validity of the CCT was the post-operative data. Infants and neonates were the most common age group suffering from complex CHDs. The mean volume of the CT dose index was 1.44 ± 0.47 mGy, the mean value of the dose-length product was 14.13 ± 5.4 mGy*cm, and the mean value of the effective radiation dose was 0.58 ± 0.13 mSv. The sensitivity, specificity, PPV, NPV, and accuracy of the low-dose prospective ECG-gated CCT for identifying complex CHDs were 95.6%, 98%, 97%, 97%, and 97% for reader 1 and 92.6%, 97%, 95.5%, 95.1%, and 95.2% for reader 2, respectively. The overall inter-reader agreement for interpreting the cardiac CCTs was good (κ = 0.74). According to the results of our investigation, low-dose prospective ECG-gated CCT is a useful and trustworthy method for assessing coronary arteries and making a precise preoperative diagnosis of complex CHDs.

## 1. Introduction

Congenital heart disease (CHD), the most common congenital disability, affects 4-50 out of every 1000 newborns [1]. CHDs can range from simple defects that affect one heart valve or a hole inside the heart, as in uncomplicated and isolated PS, VSD, ASD, and PDA, with a good prognosis, to more severe complex defects that affect several defects/anomalies of the heart, as well as the dynamics of blood circulation, and require multiple surgical correction procedures, with a doubtful prognosis [2]. With advances in imaging techniques and surgical management, 90% of CHD patients reach adulthood and have improved patient survival rates [3]. Because coronary artery anomalies are frequently associated with other CHDs, such as truncus arteriosus, tetralogy of Fallot, and a double-inlet left ventricle [4], the precise imaging identification of combined malformations in complex CHDs is crucial for accurate surgical decision planning [5,6].

Several non-invasive diagnostic modalities, such as echocardiography, magnetic resonance imaging (MRI), and computed tomography (CT), are used in the diagnosis of CHDs [7]. Although catheter angiography is a reliable method for diagnosing CHDs, its invasive nature, radiation exposure, and use of contrast medium limit its routine clinical application [8]. Currently, the diagnosis of CHDs is initiated by transthoracic echocardiography (TTE), which is non-invasive, has the ability to demarcate cardiac morphology, and allows for the Doppler-assisted measurement of flow velocities [9]. However, the main drawbacks of TTE are its operator dependence, poor ability to detect extracardiac and coronary artery anomalies, and restrictions of the acoustic window [10]. MRIs can efficiently visualize intra-and extra-cardiac structures, evaluate the function and morphology of complex CHD, and avoid the hazards of contrast medium use and ionizing radiation [11]. However, its long imaging acquisition time and need for close monitoring limit its frequent application in children [12]. Cardiac computed tomography (CCT) can evaluate intra- and extra-cardiac structures with high spatial resolution, a short scanning time, and light sedation [13]. Moreover, ECG-synchronized CCT overwhelms non-ECG-gated images by producing better quality images and detailed coronary artery evaluation [14]. Furthermore, prospective ECG-gating can lower the dose of radiation to 0.2–1.6 mSv [15]. This study aimed to define the diagnostic validity and reliability of low-dose prospective ECG-gated CCT for the preoperative evaluation of complex CHDs.

## 2. Patients and Methods

### 2.1. Ethical Statement

This study was approved by the Institutional Review Board and Research Ethics Committee of the Medical College at Najran University in the Kingdom of Saudi Arabia (approval no. 443-42-72073-DS; approved 13 July 2022). Before the study, all patients’ parents provided written informed consent. The study adhered to the ethical principles outlined in the most recent version of the Helsinki Declaration.

### 2.2. Study Population

Sixty patients with clinically suspected complex CHDs were transferred to our institution for preoperative CCT evaluation. All included patients were first subjected to TTE, which identified at least two cardiovascular anomalies in each patient.

### 2.3. Cardiac CT Acquisition 

All patients went through CCT examinations within one week prior to the operation. The scans were completed on a 128-multi-detector CT scanner (Philips Ingenuity 128) during free breathing with a low-dose prospective ECG-triggering mode. Uncooperative patients were sedated with oral chloral hydrate solution (0.1 mg/kg). For synchronization of the ECG with the CCT scan, ECG leads were connected to the electrodes after being placed at standard positions. The applied parameters were as follows: step-and-shoot axial scanning, FOV of 25 cm, collimation of 0.625 mm, rotation time of 270 ms, matrix of 512 × 512, slice thickness of 0.6 mm, temporal resolution of 135 ms, and reconstruction interval of 0.45 mm. Adjustment of the tube voltage and tube current according to patients’ body weights were performed (Table 1). Data were collected with the paddle technique using a window of 380 ms centered at 40% of the R-R interval at a heart rate of 75–140 beats/min. The scanning direction was craniocaudal and extended from the thoracic inlet level to the diaphragm in all patients. All patients received a nonionic contrast agent (Ultravist 370; Schering AG, Berlin-Wedding, Germany) (1.0–1.5 mL/kg) via an antecubital vein using a dual-head power injector at an infusion rate of 0.8–2 cc/s, followed by a 5–10 cc saline infusion at the same flow rate. Synchronization between CT scanning and contrast injection was achieved using bolus tracking. When a threshold of 100 HU was registered within the region of interest (ROI) established on the descending aorta at the level of the carina, the scan began automatically after a 7 s delay.

### 2.4. Image Post-Processing and Interpretation

Every image was sent to an external workstation (Phillips intellispace). Post-processing produced multiplanar and curved planar reformation (MPR and CPR), volume rendering (VR), and maximum intensity projection (MIP) images. All CCT images were independently interpreted by two radiologists with more than seven years of expertise in cardiac imaging who were blinded to patient clinical data and operational outcomes.

a. Evaluation of imaging quality

Subjectively

The quality of the CCT images was estimated using a five-grade scoring system [4]: grade 1, no valuable data acquired from the examination; grade 2, poor anatomical detail or image quality and incomplete delineation of the anatomical structures; grade 3, fair anatomical detail (though the definitive definition of the anatomical relationships is well depicted); grade 4, good anatomical detail and clear all structures; and grade 5, perfect anatomical detail and perfect image quality. Grades 3, 4, and 5 were sufficient for a conclusive diagnosis. The inter-reader agreement was calculated.

Objectively

The image noise and contrast-to-noise ratios (CNR) were measured on the axial images at the ascending aorta level. The image noise was the standard deviation of the pixels in a 1 cm^2^ ROI at middle aorta level. The CNRs were the division of the differences between the CT attenuation _lumen_ and CT attenuation _connective tissue_ by image noise. The CT attenuation _lumen_ was attained by putting a 12 mm^2^ ROI at the level of the ascending aorta to measure the average Hounsfield unit, and the CT attenuation _lumen_ was measured by placing a 20 mm^2^ ROI at the level of the thymus to measure the average Hounsfield unit.

b. Coronary artery evaluation

The left main coronary artery (LMCA); proximal and distal parts of the left circumflex artery (LCX); proximal, middle, and distal parts of the left anterior descending artery (LAD); and the right coronary artery (RCA) were evaluated for image quality using a five-grade scale [4]. It was interpreted as follows: Grade 1, extensive motion artifacts that hinder the visualization of the coronary segments; Grade 2, identified motion artifacts but with vague identification of the coronary segments; Grade 3, blurred visualization of the coronary segments with moderate diagnostic reliance; Grade 4, some motion artifacts but high diagnostic reliance of coronary segment delineation; and Grade 5, no motion artifacts, with a clear depiction of coronary segments. Grades 3, 4, and 5 were considered sufficient for diagnosis. All coronary segments’ diagnosis rates (of at least grade 3) and quality scores were calculated.

### 2.5. Radiation Dose Estimation

Estimations of the dose-length product (DLP) (expressed as mGy*cm), volume CT dose index (CTDI vol) (expressed as mGy), and effective dose (ED) (expressed as millisieverts (mSv)) were calculated. The scanner console identified the DLP that formulated the ED using a DLP*k equation. The conversion factor k was age-dependent and was determined using the International Commission on Radiological Protection (ICRP) publication [16].

### 2.6. Reference Standard

Two cardiothoracic surgeons, one with 12 years of experience and the other with 18, performed all operations. Operative morphologic anomaly findings were used as the standard reference for assessing the diagnostic validity of CCTs. 

### 2.7. Statistical Analysis

All statistics were performed using MedCalc 13 (MedCalc Software bvba, Ostend, Belgium), Belgium and SPSS 22.0 (SPSS Inc., Chicago, IL, USA). Quantitative data were expressed as means ± standard deviations. In the subjective image quality assessment, the inter-reader agreement (IRA) was evaluated using the kappa statistic. The κ values were interpreted as follows: 0.00–0.20, poor agreement; 0.21–0.40, fair agreement; 0.41–0.60, moderate agreement; 0.61–0.80, good agreement; and 0.81–1.00, very good agreement. To assess the diagnostic validity of the low-dose prospective ECG-triggering CCT in accurately interpreting cardiac deformities, the sensitivity, specificity, positive predictive value (PPV), negative predictive value (NPV), and accuracy were calculated based on the operative findings as the reference standards. A significant difference was interpreted for a *p*-value of ˂0.05.

## 3. Result

### 3.1. Patients and CHDs 

**Figure 1:**
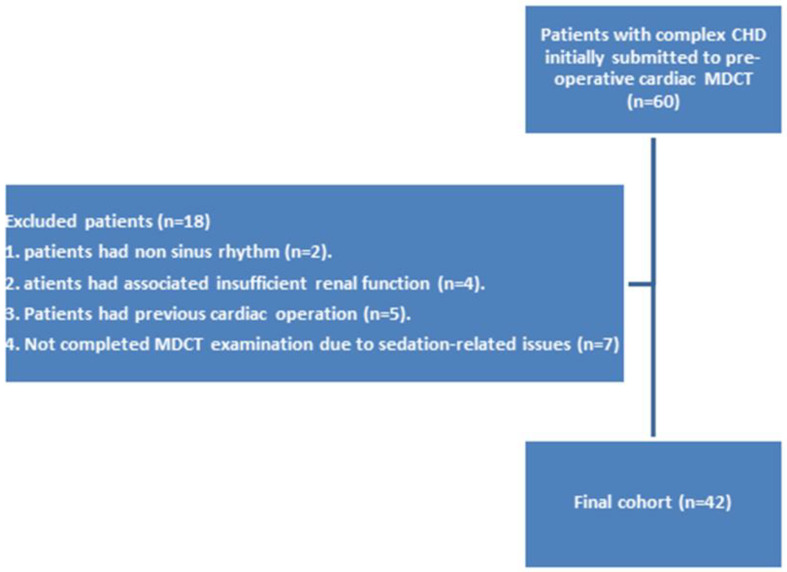
Flow chart of the study.

**Figure 2:**
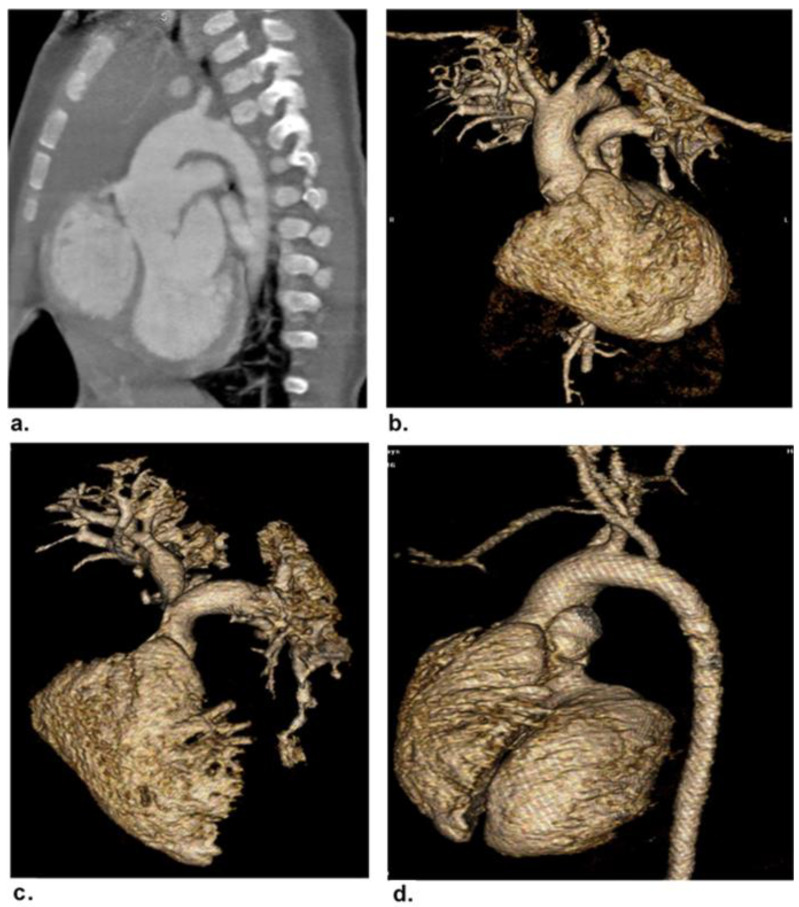
A 5-month-old male patient with truncus arteriosus Type I. (**a**) Curved MIP and (**b**) 3D-VR image showing a common arterial trunk arising with a single prominent truncal valve overriding both ventricles. The trunk measures 9 mm in length and 19 mm in diameter, along with cardiomegaly with dilatation of all chambers, predominantly right-sided, and VSD with overriding prominent truncal valve. (**c**) 3D-VR image showing a short MPA trunk arising from the left posterolateral aspect of the common trunk. This short MPA trunk measures 3.5 mm in length and 10 mm in diameter. The RPA shows a relatively tight segment (arrow) measuring approximately 2.5 mm in length and 4.5 mm in diameter at its origin. (**d**) 3D-VR image at the level of the MPA origin. The ascending aorta is seen arising from the right lateral aspect of this trunk.

**Figure 3:**
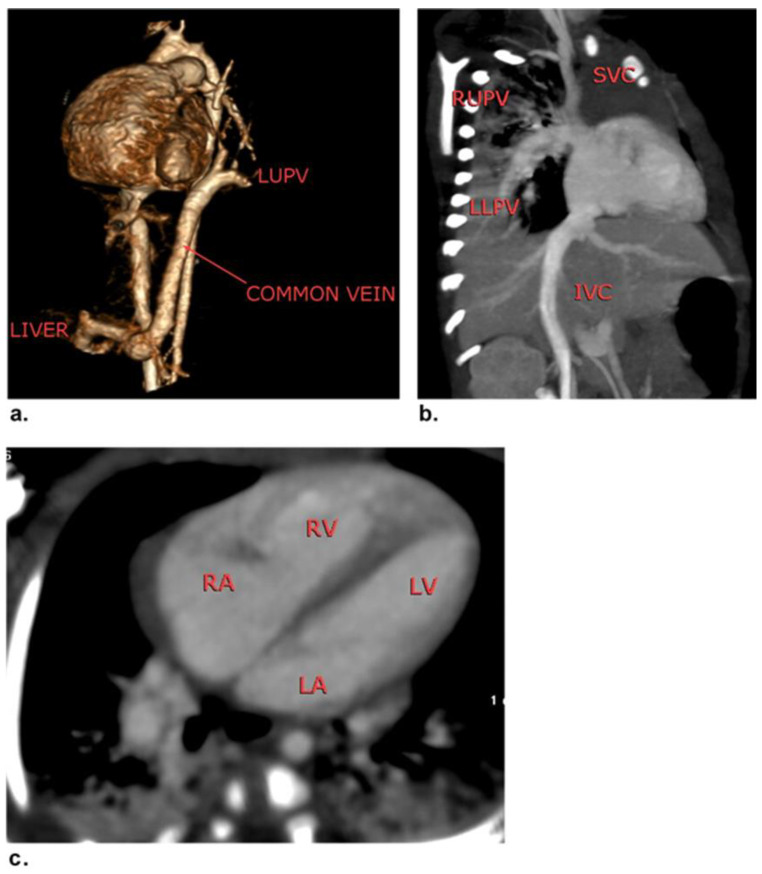
A one-month-old female patient with total anomalous pulmonary venous return (right side→SVC/left side→common vein→portal vein). (**a**) Coronal 3D-VR image revealing left upper and lower pulmonary veins communicated to form one common vein passing downward right to the aorta to join the portal vein after a 40 mm course and a 90-degree angle curve. (**b**) Curved MIP image revealing right upper and lower pulmonary veins joined at the entry site at the base of the SVC just beside the junction of a large azygous vein. (**c**) Axial MIP image revealing patent foramen ovale of 3 mm with a right to left shunt. There was no pulmonary vein connection to the left atrium.

Eighteen patients were ruled out: two had non-sinus rhythms, four had associated insufficient renal function, five had previous cardiac surgery, and seven were unable to complete the CCT examination due to sedation-related issues. The final cohort of 42 patients (20 females and 22 males; mean age of 27.3 ± 8.1 months; range of 3 days-16 years) were included in the study. Figure 1 demonstrates the flowchart of the study. The demographic and clinical features of the patients are presented in Table 2. Complex CHDs were most common in infants (45.2%) and neonates (28.6%). Table 3 shows the clinical and laboratory findings and the associated comorbidities and extracardiac malformations of our patients. The mean heart rate of our patients was 123.9 ± 20.1 beats/min (range of 75–160 beats/min). The most common presentations were chest troubles (57.1%). The most common associated comorbidities were hypertension and heart disease (9.5%). The most common associated extracardiac malformation was Down’s syndrome (11.9%). Seven patients (16.7%) had palliative surgery and 35 (83.3%) had corrective surgery. The surgical findings confirmed a total of 68 cardiovascular deformities in 42 patients. Based on the surgical findings, Table 4 shows the distribution of the complex cardiovascular deformities. The most common CHDs were extracardiac anomalies (90.1%). A respectful percentage of extracardiac and conotruncal anomalies were associated with intracardiac anomalies such as VSD and ASD (Figure 2 and Figure 3).

### 3.2. Coronary Artery Evaluation 

In the 42 CCT examinations, 378 coronary artery segments were assessed. Diagnostic rates and quality scores of the examined coronary segments were calculated (Table 5). We detected four coronary artery anomalies, including an anomalous origin in three patients and an anomalous course in one patient.

### 3.3. Diagnostic Validity of Cardiac CT 

Regarding the CCT validity for the detection of intracardiac anomalies, two false-positive cases were encountered by reader 1 and two false-negative cases were encountered by reader 2. This yielded an accuracy of 92.9%. The overall sensitivity, specificity, PPV, NPV, and accuracy of the low-dose prospective cardiac MDCT in the diagnosis of the complex CHDs were 95.6%, 98%, 97%, 97%, and 97% for reader 1 and 92.6%, 97%, 95.5%, 95.1%, and 95.2% for reader 2, respectively (Table 6).

### 3.4. Reliability of Cardiac CT 

Good to very good IRAs ere noted for the CCT evaluation of the intra-cardiac, conotruncal, abnormal connection, and extracardiac anomalies (κ = 0.77–0.94). On CCT, the overall IRA of the complex CHDs was good (κ = 0.74). The image quality IRA score was a good IRA (κ = 0.78) (Table 7).

### 3.5. Image Quality Evaluation 

Readers 1 and 2 reported excellent image quality with means of 4.45 ± 0.59 (range, 3–5) and 4.39 ± 0.57 (range, 3–5), respectively. The average CT attenuation _lumen_ was 510.54 ± 5.7 HU (range, 501–520). The average noise and CNR in the ascending aorta were 9.02 ± 2.9 HU (range, 14–23) and 26.9 ± 4.8 HU (range, 20–38), for readers 1 and 2, respectively. 

### 3.6. Radiation Dose Estimation 

The mean CTDI volume value was 1.44 ± 0.47 mGy (range, 1.07–2.8), and the mean DLP value was 14.13 ± 5.4 mGy*cm (range, 7.83–25.7). As a result, the mean effective radiation dose calculated was 0.58 ± 0.13 mSv (range, 0.38–0.95). 

## 4. Discussion

This study confirmed the high diagnostic validity of low-dose ECG-gated prospective CCTs for the preoperative evaluation of complex CHDs, with 95.2–97% overall accuracy, 92.6–95.6% sensitivity, 97–98% specificity, 95.5–97% PPV, and 95.1–97% NPV. In addition, an overall good IRA score (κ = 0.74) was obtained for the CCT interpretation. Similarly, Sigal-Cinqualbre et al. [17] reported that prospective ECG-triggering CCTs were 97.3% accurate in diagnosing cardiovascular deformities (142/146). According to Goske et al. [18], the sensitivity, specificity, PPV, and NPV of prospective ECG-triggering CCTs in diagnosing cardiovascular deformities were 94.01, 99.9, 98.6, and 99.5%, respectively.

Since children are more sensitive to X-rays and their hazards than adults [19], all ionizing radiation-based diagnostic modalities used on children must strictly adhere to the ALARA (as low as reasonably achievable) principle. As a result, radiation exposure during CCTs for children with CHDs should be given special consideration [20]. To reduce the radiation dose, modern CT scanners provide automatic dose adjustment systems, such as automated tube current modulation [21,22] and automated tube voltage selection [23,24]. In our study, we were concerned with reducing the exposure time to the radiation by adjusting tube current and voltage based on body weight, with the maximum tube current at 120 mAs and the tube voltage at 80 kV, resulting in a mean CTDI vol of 1.44 ± 0.47 mGy, a mean DLP of 14.13 ± 5.4 mGy*cm, and an estimated mean ED of 0.58 ± 0.13 mSv. Similarly, Pache et al. [12] enrolled 64 infants for MDCTs at 80 kV and 60–120 mAs, with a mean CTDIvol, DLP, and ED of 2.1 ± 0.4 mGy, 24.7 ± 5.9 mGy*cm, and 1.6 ± 0.3 mSv, respectively. In addition, Wang et al. [25] found that performing cardiac CTs at 80 kVp and 120 mAs resulted in a mean ED of 0.55 ± 0.10 mSv. In line with previous studies [26,27], we found that diagnostic quality is not impaired if automated tube-current modulation is used.

Podberesky et al. [28] reported that acquisition of CCTs by prospective triggering mode reduced the radiation dose by 73.5% (from 2.00 ± 0.35 to 0.53 ± 0.15 mSv) in comparison to the retrospective ECG-gated mode using the same kV and mAs, with a statistically significant difference (*p* < 0.001). Prospective ECG-gated CCT is based on R-wave timing, which activates the X-ray beam briefly during diastole or systole [29]. 

End-systolic reconstruction after prospective ECG-gated CCT acquisition yielded adequate thoracic and coronary artery image quality in CHDs, independent of HR, as demonstrated by Paul et al. and Sorantin et al. [30,31]. Meanwhile, Cui et al. [32] suffered from irregular HRs in prospective triggering, which prompted misregistration artifacts (stair-step artifacts). Our study did not encounter such limitations related to HR irregularities-induced artifacts, with average image quality scores of 4.45 ± 0.59 and 4.39 ± 0.57 for readers 1 and 2, respectively, and a good inter-reader agreement (k = 0.78). The mean image quality score in the coronary artery evaluation in 42 cases was 3.63 ± 0. 65. Ghoshhajra et al. [33] found that diagnostic levels of image quality could be achieved in 40–50% of the RR reconstruction images, and the mean quality scores of the CCTs and coronary artery images were 4.66 and 3.49, respectively. Because coronary artery anomalies can influence surgical management strategies, Leipsic et al. [34] ensured the accurate preoperative assessment of coronary artery anomalies in complex CHD cases.

In this study, the average attenuation in the ascending aorta was 510.54 ± 5.7 HU. In the ascending aorta, the average noise and SNR were 19.02 ± 2.9 HU and 26.9 ± 4.8, respectively. Our findings are on agreement with Gao et al. [29], who measured 15.75 ± 3.61 HU for mean noise and 28.19 ± 13.00 HU for CNR.

In our study, we encountered some CT artifacts as streak artifacts, which were blamed for the false-positive CCT results that were misinterpreted as atrial septal defects caused by high-attenuation contrast media in the right atrium. The false-negative results, on the other hand, were caused by a small atrial septal defect (3 mm) and a small patent ductus arteriosus. Pache et al. [12] stated that atrial septal defect reporting should be performed with caution because of the thinning nature of the interatrial septum, which makes it difficult to be delineated on CT examinations, particularly at the fossa ovalis.

The fact that prospective ECG-triggering CCT is less impacted by respiratory motion makes it more practical for use in young children who are breathing on their own [35]. There were no unsuccessful scans due to respiratory movements in this research. 

The mean heart rate of our patients was 123.9 ± 20.1 beats/min. The data in our study were collected with the paddle technique using a window of 380 ms, centered at 40% of the R-R interval at a heart rate of 75–140 beats/min. This scanning mode allows a satisfactory image to be acquired in newborns and infants with very high heart rates.

In our study, we found that the most common associated comorbidities were hypertension and heart disease (9.5%). The most common associated extracardiac malformation was Down’s syndrome (11.9%). In our study, the rates of comorbidities and extracardiac malformations, along with CHDs, were nearly similar to those of other studies [36,37]. 

The current study had several limitations. First, no comparisons were made between the use of CCT and other imaging techniques such as cardiac magnetic resonance imaging (CMR). Second, the CCTs did not provide functional or hemodynamic information, such as wall motion abnormalities. Fourth, the small sample size resulted in a wide confidence interval. To confirm and extend our findings, larger sample size comparative studies are required.

## 5. Conclusions

In conclusion, low-dose prospective ECG-gated CCTs have high diagnostic validity and reliability in the preoperative diagnosis of complex CHDs and coronary artery evaluations, with the added value of radiation dose reduction and good image quality. However, the decision for pre-operative imaging, including the use of CT angiography, continues to be best determined by the clinical management team

## Figures and Tables

**Table 1:** Low-dose prospective ECG-gated cardiac CT parameters with weight-based radiation dose adjustments.

Body Weight (kg)	Tube Voltage (kV)	Tube Current (mAs)
0–3	80	60
3.1–6	80	80
6.1–10	80	100
>10	80	120

ECG = electrocardiography; CT = computed tomography.

**Table 2:** Demographic data of the patients.

Variable	Value
Age, months	27.3 ± 8.1 (3 days–16 years)
Age groups, number (%) Neonates (1–30 days) Infants (31 days–2 years) Preschool children (2–6 years) School children (6–12 years) Adolescents (>12 years)	12 (28.6) 19 (45.2) 5 (11.9) 4 (9.5) 2 (4.8)
Sex, number (%)	
Male	22 (52.4)
Female	20 (47.6)

Note: unless otherwise indicated, the data represent means ± SDs, with ranges in parentheses.

**Table 3:** Clinical and laboratory findings and associated comorbidities and malformations.

Variable	Value
Clinical Weight (gm), mean ± SD (range) Heart rate (beat/min), mean ± SD (range) Cyanosis (%) Chest troubles (%) Dyspnea (%) Delayed milestone (%) Edema (%) Ascites (%) Laboratory Hemoglobin (g/dL), mean ± SD Hematocrit, mean ± SD Cholesterol, (mg/dL), mean ± SD Glucose, (mg/dL), mean ± SD C-reactive protein, (mg/dL), mean ± SD Associated comorbidities and extracardiac malformations Hypertension (%) Heart disease/ischemic disease (%) Obesity (%) Diabetes (%) Dyslipidemia (%) Pneumonia (%) Down’s syndrome (%) Cleft lip or Cleft palate (%) Hirschprung disease (%) Esophageal atresia (%) Mental retardation (%) Scoliosis (%) Renal dysplasia (%)	10,026.2 ± 1085.1 (1400–45,000) 123.9 ± 20.1 (75–160) 17 (40.5) 24 (57.1) 9 (21.4) 15 (35.7) 7 (16.7) 4 (9.5%) 13.1± 1.5 38.9 ± 3.1 145.4 ± 51.8 81.2 ± 10.2 0.2 ± 0.1 4 (9.5) 4 (9.5) 3 (7.1) 3 (7.1) 2 (4.8) 3 (7.1) 5 (11.9) 2 (4.8) 1 (2.4) 2 (4.8) 2 (4.8) 1 (2.4) 1 (2.4)

**Table 4:** CHDs in 42 patients.

CHDs	Number of Patients (%)
Intracardiac/Cardiac Malposition Ventricular septal defect Atrial septal defect Dextrocardia	8 (11.8) 6 (8.8) 2 (2.9)
Conotruncal Tetralogy of Fallot Truncus arteriosus	7 (10.3) 3 (4.4)
Abnormal Connection Transposition of great arteries Double outlet right ventricle	2 (2.9) 2 (2.9)
Extracardiac Total anomalous pulmonary venous return Aortic coarctation Patent ductus arteriosus Double aortic arch Supravalvular aortic stenosis Pulmonary artery stenosis Pulmonary atresia Coronary artery anomalies	4 (5.9) 6 (8.8) 10 (14.7) 1 (1.5) 3 (4.4) 7 (10.3) 3 (4.4) 4 (5.9)
Total	68

CHDs = congenital heart diseases.

**Table 5:** Diagnostic rates and image quality scores for all coronary segments.

	Diagnostic Rate	Quality Score Mean ± SD (Range)
LMCA	42	4.47 ± 0.5 (4–5)
LAD Proximal Mid Distal	42 40 36	4.42 ± 0.5 (4–5) 3.6 ± 0.9 (1–5) 2.7± 0.8 (1–4)
LCX Proximal Distal	42 30	4.19 ± 0.55 (3–5) 2.3 ± 0.9 (1–4)
RCA Proximal Mid Distal	42 35 31	3.78 ± 0.6 (3–5) 2.8 ± 0.7 (1–4) 2.8 ± 0.8 (2–4)
Overall	42	3.63 ± 0. 65 (2.2–4.8)

SD = standard deviation; LMCA = left main coronary artery; LAD = left anterior descending artery; LCX = left circumflex artery; RCA = right coronary artery.

**Table 6:** Diagnostic validity of the low-dose prospective ECG-triggering cardiac CT in diagnosing complex CHDs.

	Reader 1	Reader 2
Intracardiac/cardiac malposition anomaly		
Accuracy %	92.9 [80.5–98.5]	92.9 [80.5–98.5]
Sensitivity %	93.8 [69.7–99.8]	87.5 [61.6–98.5]
Specificity %	92.3 [74.9–99.1]	96.2 [80.4–99.9]
PPV %	88.2 [66.3–96.6]	93.3 [67–98.9]
NPV %	96 [78.2–99.3]	92.6 [77.3–97.9]
AUC	0.93	0.91
Conotruncal anomaly		
Accuracy %	100 [91.6–100]	95.2 [83.8–99.4]
Sensitivity %	100 [69.2–100.0]	90 [55.5–99.7]
Specificity %	100 [89.1–100]	96.9 [83.8–99.9]
PPV %	100	90 [56.4–98.4]
NPV %	100	96.9 [82.3–99.5]
AUC	1	0.93
Abnormal connection anomaly		
Accuracy %	100 [91.6–100]	95.2 [83.8–99.4]
Sensitivity %	100 [39.8–100]	75 [19.4–99.4]
Specificity %	100 [90.7–100]	97.4 [86.2–99.49
PPV %	100	75 [28.6–95.7]
NPV %	100	97.3 [87.1–99.5]
AUC	1	0.86
Extra-cardiac anomaly		
Accuracy %	95.2 [83.8–99.4]	97.6 [87.4–99.9]
Sensitivity %	94.7 [82.2–99.4]	97.4 [86.2–99.9]
Specificity %	100 [39.8–100]	100 [39.8–100]
PPV %	100	100
NPV %	66.7 [34.2–88.5]	80 [36.6–96.5]
AUC	0.97	0.99
Overall		
Accuracy %	97 [93.2–99]	95.2 [90.8–97.9]
Sensitivity %	95.6 [87.6–99.1]	92.6 [83.7–97.6]
Specificity %	98 [92.9–99.8]	97 [91.5–99.4]
PPV %	97 [89.2–99.2]	95.5 [87.3–98.5]
NPV %	97 [91.5–98.9]	95.1 [89.3–97.8]
AUC	0.97	0.95

Note: the data in brackets are 95% confidence intervals. CHDs = congenital heart diseases; CT = computed tomography; PPV = positive predictive value; NPV = negative predictive value; AUC = area under curve.

**Table 7:** Inter-reader agreement for the cardiac CT interpretation.

Variable	Value
Intra-cardiac anomaly	0.89 (0.76–1.00)
Conotruncal anomaly	0.94 (.81–1.00)
Abnormal connection anomaly	0.88 (0.64–1.00)
Extra-cardiac anomaly Image quality	0.77 (0.48–1.00) 0.78 (0.51–1.00)

Note: The data are expressed in Kappa values, and the data in parentheses are 95% confidence intervals. The κ values were interpreted as follows: 0.00–0.20 = poor agreement; 0.21–0.40 = fair agreement; 0.41–0.60 = moderate agreement; 0.61–0.80 = good agreement; and 0.81–1.00 = very good agreement.

## Data Availability

The datasets used and/or analyzed during the current study are available from the corresponding author on reasonable request.
